# Krankheitsbezogener Wissenserwerb durch strukturierte Patienteninformation bei Rheumatoider Arthritis (StruPI-RA): Erste Ergebnisse der StruPI-RA-Studie in Deutschland

**DOI:** 10.1007/s00393-020-00871-7

**Published:** 2020-09-14

**Authors:** M. Schwarze, V. Fieguth, F. Schuch, P. Sandner, E. Edelmann, A. Händel, M. Kettler, A. Hanke, M. Kück, L. Stein, C. Stille, M. Fellner, V. De Angelis, S. Touissant, C. Specker

**Affiliations:** 1 Institut für Sportmedizin, Medizinische Hochschule Hannover, Carl-Neuberg-Str. 1, 30625 Hannover, Deutschland; 2 Rheumatologische Schwerpunktpraxis, Erlangen, Deutschland; 3 Rheumazentrum Bad Aibling-Erding, Bad Aibling, Deutschland; 4 Rheumatologie-Praxis, Hannover, Deutschland; 5 Rheumatologie Centrum, Leverkusen, Deutschland; 6 Klinik für Rheumatologie & Klinische Immunologie, Kliniken Essen-Mitte, Essen, Deutschland; 7 Arbeitsgemeinschaft Regionaler Kooperativer Rheumazentren in der Deutschen Gesellschaft für Rheumatologie e. V. (DGRh), Berlin, Deutschland

**Keywords:** Gesundheitskompetenz, Patientenschulung, Patient Knowledge Questionnaire, Frühe Rheumatoide Arthritis, Ambulante Versorgung, Health Literacy, Patient education, Patient knowledge questionnaire, Early rheumatoid arthritis, Outpatient care

## Abstract

**Hintergrund/Ziel:**

Mit der strukturierten Patienteninformation für Rheumatoide Arthritis (StruPi-RA) liegt das erste standardisierte ambulante Patientenschulungsprogramm für Rheumatoide Arthritis (RA) in Deutschland vor. Das Hauptziel der vorliegenden Studie ist die Erfassung der Wirksamkeit von StruPI-RA in Bezug auf den krankheitsspezifischen Wissenserwerb bei Patienten mit früher RA oder nach Therapiewechsel.

**Methoden:**

Insgesamt wurden 61 Patienten eingeschlossen: *n* = 32 in die Interventionsgruppe (IG) und *n* = 29 in die Kontrollgruppe (KG). Die Intervention umfasste ein strukturiertes Patienteninformationsprogramm (StruPi-RA) zu den Themen Diagnostik, Therapie und Leben mit RA, welches drei 90-minütige Module beinhaltet. Die KG erhielt nur einen Patientenratgeber der Deutschen Rheumaliga zur Information über die Erkrankung. Primäres Zielkriterium war der krankheitsbezogene Wissenserwerb, der anhand des Patient Knowledge Questionnaire (PKQ) sowie Erweiterungsfragen zu 2 Messzeitpunkten, einmal unmittelbar vor und dann nach Durchführung von StruPI-RA, erhoben wurde.

**Ergebnisse:**

Die Teilnahme an StruPI-RA führte zu einer signifikanten Verbesserung des krankheitsspezifischen Wissens im Gruppen- und Zeitvergleich zur ungeschulten Gruppe im Original-PKQ sowie in der Fragebogenerweiterung. Ein Einfluss der Krankheitsdauer oder des Bildungsstands wurde nicht beobachtet. Allein in der Subskala Therapie zeigte sich ein signifikanter Unterscheid im Gruppen- und Zeitvergleich.

**Diskussion:**

Die Teilnehmer am StruPI-RA-Programm hatten im Vergleich zu ungeschulten Patienten einen nachweisbaren krankheitsspezifischen Wissenszuwachs. Dadurch kann sich die Arzt-Patienten-Kommunikation verbessern und eine fundierte Entscheidungsfindung hinsichtlich der Therapie befördert werden. Mittelfristig können sich darüber hinaus eine erhöhte Selbstmanagementkompetenz der Patienten und langfristig auch eine Verbesserung der Lebensqualität sowie der Therapieadhärenz ergeben.

**Zusatzmaterial online:**

Die Online-Version dieses Beitrags (10.1007/s00393-020-00871-7) enthält einen Fragenkatalog und die Rohdaten zu Abb. 2. Beitrag und Zusatzmaterial stehen Ihnen auf www.springermedizin.de zur Verfügung. Bitte geben Sie dort den Beitragstitel in die Suche ein, das Zusatzmaterial finden Sie beim Beitrag unter „Ergänzende Inhalte“.

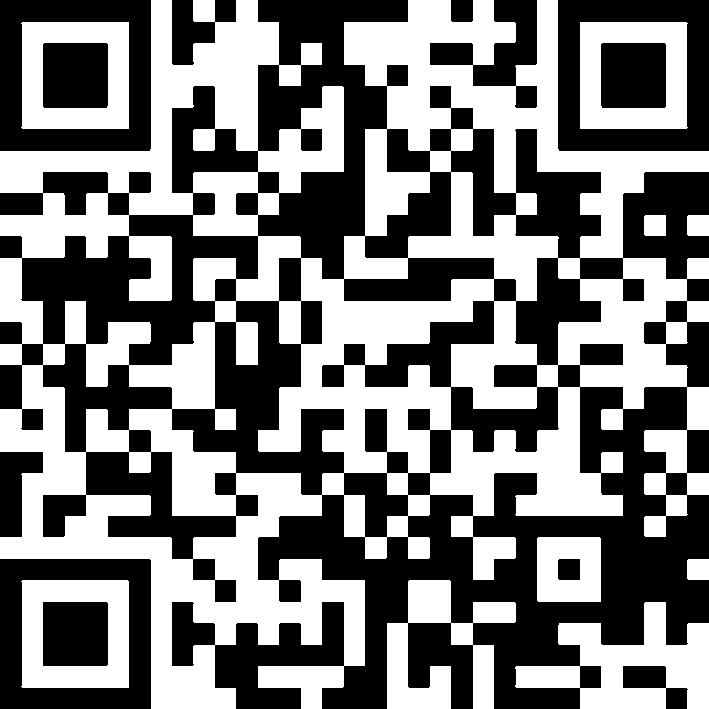

## Abstract (en)

**Background/objective:**

The structured patient information for rheumatoid arthritis (StruPi-RA) program was the first standardized outpatient education program in rheumatoid arthritis (RA) in Germany. The main objective of the study was to determine the efficacy of the StruPi-RA program concerning disease-specific knowledge acquisition in patients with early stage RA or after changing the treatment regimen.

**Methods:**

A total of 61 patients were included in a control group design, 32 in the intervention group (IG) and 29 in the control group (CG). Patients of the IG attended 3 modules of 90 min in a structured patient information program (StruPI-RA) including the topics of diagnostics, treatment and living with RA. Patients in the CG only received information material from the German Rheumatism League. The primary target criterion was the disease-related acquisition of knowledge, measured with the patient knowledge questionnaire (PKQ). Data were collected before and after participation in StruPI-RA.

**Results:**

The improvement in knowledge in the IG attending the StruPI-RA compared to the CG was significant in time and group comparisons. No influence of disease duration or educational level was observed. The subscale treatment alone showed a significant difference in the group and time comparison.

**Conclusion:**

Participation in the StruPI-RA program in early RA was associated with a significant increase in disease-specific knowledge compared to the control group of patients. This leads to better decision-making in terms of treatment, a more beneficial doctor-patient communication and better self-management. In the long term an improvement in treatment adherence and quality of life is expected.

## Hintergrund und Fragestellung

### Aktuelle Prävalenz, Krankheitsbild und Therapiestandards

Die Rheumatoide Arthritis (RA) ist mit einer geschätzten Prävalenz je nach Falldefinition von 0,6–1,4 % in der erwachsenen deutschen Bevölkerung die häufigste chronisch entzündliche Gelenkerkrankung [17]. Dabei sind Frauen doppelt so häufig betroffen wie Männer, der Altersgipfel bei Erkrankungsbeginn liegt bei ca. 50 Jahren. Das Krankheitsbild geht unbehandelt mit schmerzhaft geschwollenen Gelenken, einer fortschreitenden Gelenkzerstörung einher und kann zu erheblicher Morbidität, z. B. durch Funktionseinbußen, Mobilitätseinschränkungen, einer erhöhten Rate von kardiovaskulären Ereignissen und Neigung zu Depressionen führen [6, 7]. Freier et al. [7] stellten fest, dass bereits bei Früharthritispatienten im Vergleich zur Normalbevölkerung eine höhere Prävalenz von depressiven und ängstlichen Symptomen vorliegt. Neben Funktionseinschränkungen und Schmerzen sind die Patienten von einer deutlich eingeschränkten Lebensqualität betroffen [15].

In den letzten 10 bis 20 Jahren haben sich die Prognose und der Krankheitsverlauf der RA erheblich verbessert. Zum einen durch neue serologische Biomarker (anticitrullinierte Peptidantikörper [ACPA]), zum anderen durch ein deutlich größeres Armamentarium zielgerichteter, effektiver medikamentöser Therapien [11, 26]. So lässt sich eine RA heute bereits in früheren Krankheitsphasen als zuvor diagnostizieren, was einen frühen Therapiebeginn ermöglicht, der auch ein besseres Outcome der Patienten zur Folge hat. Inzwischen ist somit das Ziel der Behandlung bei der RA die Remission, also die Abwesenheit von jeglicher Krankheitsaktivität, was die Wahrscheinlichkeit des Ausbleibens von erkrankungsbedingten Spätfolgen erheblich steigert [26]. Die medikamentöse Therapie erfolgt nach aktueller S2e-Leitlinie der Deutsche Gesellschaft für Rheumatologie nach einem speziellen Algorithmus nach dem Prinzip des „Treat-to-Target“ (T2T) [4, 11, 12].

Die frühe Diagnose wird auch durch die American College of Rheumatology (ACR) und der European League Against Rheumatism(EULAR)-Klassifikationskriterien von 2010 erleichtert. Eine frühzeitige Einleitung der medizinischen Behandlung mit Disease-modifying anti-rheumatic drugs (DMARDs) innerhalb von 3 Monaten wird empfohlen, um das Fortschreiten der Erkrankung zu verhindern und damit die Langzeitprognose zu verbessern [26, 28].

In der deutschen Versorgungslandschaft bestehen allerdings noch Defizite bei der Umsetzung dieser zielorientierten Therapie unter Alltagsbedingungen. Laut Kerndokumentation der regionalen kooperativen Rheumazentren ist nur etwas über ein Drittel der Patienten in Remission, knapp über die Hälfte weist eine moderate oder hohe Krankheitsaktivität auf [2]. Die Course and Prognosis of Early Arthritis(CAPEA)-Studie zeigte zwar, dass 40 % der Patienten mit früher RA innerhalb der ersten 6 Monate eine Remission erreichen, nach diesem Zeitraum erfolgen aber wenig Therapieanpassungen, um auch bei den anderen Patienten eine Remission zu erzielen [1].

### Zusammenhänge zwischen Adhärenz/Gesundheitskompetenz und Behandlungsergebnis

In der Umsetzung der Behandlungsstandards für die RA ist zu beobachten, dass die im Arzt-Patienten-Kontakt getroffenen Entscheidungen in Bezug auf die Medikation nicht immer bzw. nur teilweise im tatsächlichen Verhalten der Patienten umgesetzt werden. So lag die Adhärenz hinsichtlich der medikamentösen Therapie nur zwischen 30 und 80 % [21]. In der Literatur haben die Krankheitsschwere und die Art der Medikation keinen Einfluss auf die Therapieadhärenz, und Untersuchungen zur Bedeutung sozioökonomischer Faktoren auf die Therapieadhärenz liefern uneinheitliche Ergebnisse. Demgegenüber übt eine gute Arzt-Patienten-Beziehung einen günstigen Einfluss auf die Therapieadhärenz aus wie auch Krankheitswissen, Selbstwirksamkeit und gesundheitliche Überzeugungen auf Patientenseite [21, 29]. Als eine Ursache für geringe Adhärenz stellten Galo et al. [13] einen Mangel an Verständnis hinsichtlich Diagnostik und medizinischer Behandlung seitens der Patienten fest. In einer großen Beobachtungsstudie mit 708 RA-Patienten in Deutschland kamen Kuipers et al. [18] zu dem Ergebnis, dass Adhärenz und Gesundheitskompetenz zwar einen geringen, aber konsistenten Einfluss auf die Verbesserung des Therapieergebnisses haben. Eine Forschergruppe um Fayet et al. [9] beobachtete insbesondere bei älteren und bildungsfernen Patienten ein niedrigeres Krankheitswissen und stellte fest, dass eine Erhöhung des krankheitsbezogenen Wissens mit einem verbesserten Behandlungserfolg einhergeht.

### Bedeutung von Patientenschulungen

Zur Bewältigung einer rheumatischen Erkrankung ist die Beteiligung der Patienten an der Therapieplanung und die Stärkung der Gesundheitskompetenz eine wichtige Aufgabe [4, 26]. In den EULAR-Empfehlungen wird die partizipative Entscheidungsfindung („shared decision making“) als integraler Bestandteil der Behandlung innerhalb einer koordinierten Versorgung von Patienten mit RA gefordert. Eine Patientenaufklärung, -information und -schulung sollte den Patienten in allen Phasen der rheumatischen Erkrankung ermöglicht werden [26]. Mit der Übersetzung und Adaption der EULAR-Empfehlungen für den deutschsprachigen Raum wurde durch die DGRh ebenfalls auf die elementare Bedeutung von Patientenschulungen als Teil des optimalen Managements rheumatischer Erkrankungen hingewiesen [22]. Aktuell wurden die rheumatologischen Schulungsprogramme aktualisiert und ein Rahmenkonzept erstellt, welches einen flexiblen Einsatz der Programme ermöglicht [24, 25].

Um den Einfluss einer Patientenschulung auf den Krankheitsverlauf belegen zu können, müssen die Schulungs- und Informationsangebote sowie deren Auswirkungen z. B. auf Patientenwissen und -verhalten mit entsprechenden Instrumenten evaluiert werden. Nur dann ist mit einer breiten Akzeptanz einer Patientenschulung bei den Betroffenen, den Anbietern und den Kostenträgern zu rechnen. Für die empirische Absicherung werden als Outcome-Parameter dieser ersten Evaluation von StruPI-RA die Ziele des Programms in Bezug auf die Wissensvermittlung in den Fokus genommen.

### Entwicklung des StruPI-RA-Konzeptes

In Deutschland waren Patientenschulungen bislang überwiegend auf den stationären Bereich beschränkt. Diese Schulungen werden v. a. im Rahmen von Reha-Maßnahmen durchgeführt und kommen somit eher im späteren Krankheitsverlauf zum Einsatz. Ambulant sind solche Schulungskonzepte durch ihre Komplexität und die Notwendigkeit der Einbindung verschiedener „Trainer“ in der Regel kaum durchführbar. Die Behandlung der meisten Patienten mit RA erfolgt jedoch im ambulanten Bereich. Daher wurde für ambulante Patienten mit RA von der Arbeitsgemeinschaft der regionalen kooperativen Rheumazentren (AGRZ), dem Berufsverband Deutscher Rheumatologen (BDRh) und der Deutschen Rheumaliga (DRL) gemeinsam ein kürzeres, alltagstaugliches interaktives Schulungsprogramm, „StruPI-RA“, entwickelt, welches sich auf Patienteninformation konzentriert. Zur Durchführung ist hierbei neben einem Rheumatologen die Einbindung einer rheumatologischen Fachassistenz (RFA) gefordert, welche seit einigen Jahren eine neue, fachlich fundierte, aber niederschwellige und mehr auf der Patientenebene agierende Versorgungsebene in rheumatologischen Praxen und Kliniken darstellt. Ein Schulungsteam für StruPI-RA, bestehend aus einem Rheumatologen und mindestens einer RFA, muss vor Durchführung der Schulung ein ganztägiges „Train-the-Trainer“-Seminar absolvieren, in dem neben den Inhalten auch didaktische und lehrpädagogische Techniken und Instrumente vermittelt werden. Für die Durchführung der 3 StruPI-RA-Module stehen begleitende Lehr- und Lernmaterialien sowohl für Patienten als auch für die Referenten in Form von Manuals und Präsentationen mit PowerPoint der Firma Microsoft Cooperation zur Verfügung [20].

Das strukturierte Patienteninformationsprogramm „StruPI-RA“ soll Patienten zu einem frühen Zeitpunkt nach Diagnosestellung oder bei einem Therapiewechsel angeboten werden. Der Informationsbedarf und die zu erlernende Krankheitsbewältigung („Coping“) für Patienten mit der Erstdiagnose einer chronischen, in der Regel lebenslangen Erkrankung ist erheblich. Schmerzen, Bewegungseinschränkung, Erschöpfung, negative Emotionen und Ängste beeinflussen Patienten nachhaltig in sämtlichen Lebensbereichen: Privat- und Familienleben, Sexualität, Ausbildung, Beruf und sonstige gesellschaftliche Teilhabe. Hier setzt StruPI-RA an, um über die Entstehung der Erkrankung, die vielen Therapieoptionen, medikamentös, nichtmedikamentös und weitere Aspekte, z. B. Sozialmedizin, Selbsthilfe und Ernährung, zu informieren und zu beraten. StruPI-RA zielt darauf ab, das gesundheitsorientierte Verhalten der Patienten durch strukturierte Informationen zu verbessern. Obwohl die Patienten*information* den Hauptbestandteil von StruPI-RA bildet, werden, darauf aufbauend, den Teilnehmern auch Verhaltensänderungen und Anpassungsstrategien vermittelt, welche sie in einem besseren, selbstständigen Krankheitsmanagement *schulen*.

### Eigene Vorstudien

In eigenen Vorstudien der Autorengruppe zeigten Experteninterviews, dass StruPI-RA sowohl für die Patienten als auch für die Ärzte und RFA hilfreich und von potenziellem Nutzen ist. Mithilfe des „Austrian-German Educational Needs Assessment Tool-Fragebogens“ (OENAT) [19] wurde der Informationsbedarf zu den Themengebieten Therapie der Rheumatoiden Arthritis, Umgang mit Schmerzen, Gefühlen, angemessene Bewegung und Selbsthilfe bei RA-Patienten erhoben [10]. Von über 100 Patienten gaben 73 % Interesse an gezielten Informationen zum Umgang mit der Erkrankung an, und 80 % gaben an, gerne an StruPI-RA teilnehmen zu wollen [10, 20, 27]. Patienten wünschten sich dabei, gezielt Fragen stellen zu können, glaubten, sich in der Gruppe nicht alleine zu fühlen, und meinten, so besser mit ihrer Krankheit umgehen zu können. Die Praxen und Ambulanzen, die StruPI-RA bereits eingeführt haben, können dadurch direkt einer ganzen Gruppe von Patienten und deren Angehörigen in überschaubarer Zeit Informationen zu der Erkrankung und zu Verhaltensweisen in der Therapie- und Krankheitskontrolle vermitteln, und sie bekommen über die Diskussion in der Gruppe eine direkte Rückkopplung zum Verständnis, zu adäquater „Informationstiefe“ und evtl. bestehenden Lücken im Wissen über oder Verhalten mit der Erkrankung RA. Die StruPI-RA-Schulungsteams sind von dem Programm überzeugt und möchten in ihren Einrichtungen weiterhin regelmäßig schulen.

### Herleitung der Fragestellung

Der krankheitsspezifische Wissenserwerb stellt eine Basis für die Entwicklung von Gesundheitskompetenz und dem Treffen einer informierten Entscheidung dar. Diese sog. proximalen Ziele sind wiederum eine wichtige Voraussetzung für das Erreichen der distalen Ziele wie Krankheitsbewältigung, Förderung der Lebensqualität und Therapieadhärenz.

Beantwortet werden soll die Frage, ob RA-Patienten von einer strukturierten, ambulanten Patienteninformation mit StruPI-RA gegenüber Patienten mit gleicher Indikation und vergleichbaren Voraussetzungen ohne die entsprechende Intervention im Hinblick auf den Wissenserwerb profitieren. Hierbei sollen zunächst die kurzfristigen Effekte nach der StruPI-RA-Schulung auf der Ebene des krankheitsspezifischen Wissenserwerbs evaluiert werden.

## Studiendesign und Untersuchungsmethoden

Die Studie wurde in einem multizentrisch kontrollierten Prä-Post-Studien-Design durchgeführt. Patienten mit einer gesicherten frühen RA-Diagnose nach den Klassifikationskriterien des American College of Rheumatology (ACR) und der European League Against Rheumatism (EULAR) [3] oder nach einem Therapiewechsel innerhalb der letzten 6 Monate wurden in die Studie einbezogen. Die Patienten mussten an allen 3 Modulen von StruPI-RA teilgenommen haben, die Trainingsinhalte verfolgen und den Fragebogen selbstständig ausfüllen können. Die Patienten der IG wurden von 4 rheumatologischen ambulanten Praxen nacheinander für die Teilnahme an der Studie rekrutiert. Die KG wurde ausschließlich von einer Praxis gestellt.

### Intervention

Die Intervention StruPI-RA hat das Ziel, krankheitsspezifisches Wissen zu vermitteln und die Krankheitsbewältigung in der frühen Phase der RA oder bei Therapiewechsel zu verbessern. Die Trainingseinheiten im Rahmen von StruPI-RA umfassen drei 90-minütige Module über einen Zeitraum von maximal 3 Wochen (s. Tab. 1).

**Table 1:** 

*Modul 1: Krankheitsbild und Diagnose*	Ursachen
	Symptome
	Untersuchungen RA
*Modul 2: Therapie*	Überwachung der Krankheitsaktivität
	Behandlung und Kenntnis der medikamentösen Behandlung („disease-modifying anti-rheumatic drugs“ [DMARDs])
	Vor- und Nachteile bestimmter Therapien
	Notwendige Kontrollen
*Modul 3: Leben mit RA*	Umgang mit der Krankheit im Alltag und bei der Arbeit
	Rolle des Partners und des sozialen Umfelds
	Spezielle Fragen wie: Behinderungen oder Schwangerschaft

Rheumatologen und RFA leiten die einzelnen Sitzungen gemeinsam. Für den Zeitraum der 3 Module bleiben die Gruppen geschlossen, und die Gruppengröße des Trainings ist auf 12 Patienten begrenzt, um eine gute Interaktion zwischen den Teilnehmern und Seminarleitern zu gewährleisten. Für Ärzte und RFA wurden zuvor Train-the-Trainer-Seminare angeboten, um sich mit Materialien und didaktischen Methoden für den Gruppenunterricht vertraut zu machen.

### Datenerfassung

Die Datenerfassung erfolgte zu 2 Messzeitpunkten. Die Teilnehmer der IG füllten die Fragebögen für den Messzeitpunkt t1 (unmittelbar vor StruPI-RA) und zum Messzeitpunkt t2 (unmittelbar nach StruPI-RA) in der durchführenden rheumatologischen Einrichtung aus. Die Patienten der KG erhielten nach dem Ausfüllen des Fragebogens für t1 eine ausführliche schriftliche Broschüre der Deutschen Rheuma Liga (DRL) über das Krankheitsbild [5]. Das Ausfüllen der Fragebögen t2 erfolgt ebenfalls in der Praxis/Ambulanz. Ergänzt werden medizinische Angaben zur Krankheitsaktivität (Disease Activity Score 28). In dem Evaluationsprojekt zu StruPI-RA werden zu weiteren Zeitpunkten Patientenbefragungen durchgeführt und medizinische Daten erhoben, die aber nicht Bestandteil dieser (ersten) Analyse sind.

### Messinstrumente

**Figure 1:**
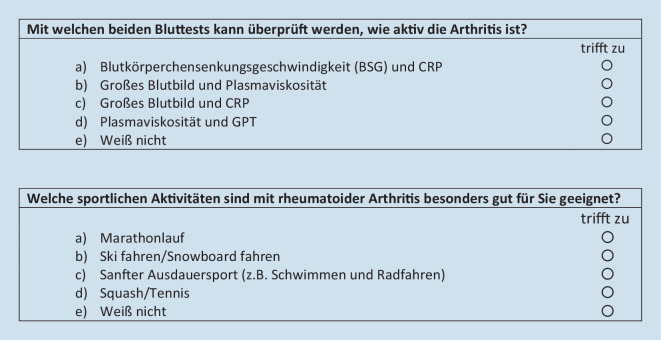

Der Patient Knowledge Questionnaire (PKQ) ist ein valides und sensitives Instrument zur Messung des Wissenserwerbs zur Rheumatoiden Arthritis und wurde an Patienten mit früher RA vor und nach einer Patienteninformationsschulung getestet [16]. Der PKQ besteht aus 12 Multiple-Choice-Fragen mit jeweils 5 Antworten (s. Beispielfragen in Abb. 1).

Themenbereiche sind unter anderem Ätiologie, Zeichen und Symptome, Arzneimitteltherapie und -überwachung, Gelenkschutz und -bewegung sowie ressourcenschonendes Verhalten. Der PKQ wurde bei einer inhaltlich und zeitlich ähnlichen Patientenschulung wie StruPI-RA zur Erfassung des krankheitsspezifischen Wissenserwerbs bei Patienten mit früher RA verwendet. Die originale englischsprachige Fassung wurde in die deutsche Sprache übersetzt und die Verständlichkeit in einem Pretest (*n* = 5) überprüft.

Um die Module „Krankheitsbild und Diagnose“, „Therapie“ und „Leben mit RA“ der StruPI-RA-Intervention umfassend hinsichtlich des krankheitsbezogenen Wissenserwerbs abzudecken, wurde der PKQ-Fragebogen („PKQ-Original“) um 6 analoge Multiple-Choice-Fragen zur körperlichen Aktivität sowie zur Bewältigung von Arbeit und Alltag erweitert („Erweiterung“) (s. Tab. 2 und Zusatzmaterial online).

**Table 2:** 

	Krankheitsbild und Diagnose	Therapie	Leben mit RA
PKQ-Original (12 Fragen)	Fragen 2, 3, 5	Fragen 6, 7, 8, 9, 10, 11	Fragen 14, 15, 16
Erweiterung (6 Fragen)	Fragen 1, 4	Fragen 12, 13	Fragen 17, 18

*RA* Rheumatoide Arthritis

### Statistische Auswertung

Die Ergebnisse sind als Mittelwert und Standardabweichung angegeben. Die Normalverteilung wurde mit dem Kolmogorov-Smirnov-Test überprüft. Mittelwertunterschiede zwischen der IG und der KG wurden für normalverteilte Daten mit einem zweiseitigen, ungepaarten t‑Test und für nichtnormalverteilte mit einem Mann-Whitney-*U*-Test mit Cohen’s d als Effektstärke berechnet. Unterschiede zwischen den Messzeitpunkten t1 und t2 innerhalb der beiden Gruppen wurden für normalverteilte Daten mit einem zweiseitigen, gepaarten t‑Test und für nichtnormalverteilte mit einem Wilcoxon-Test mit Hedge’s g als Effektstärke überprüft. Für die Effektstärken gelten ein kleiner Effekt ab 0,2, ein mittlerer ab 0,5 und ein großer ab 0,8. Der Gruppen- und Zeitvergleich zwischen den Messzeitpunkten und den Gruppen wurde mittels einfaktoriellen Varianzanalysen mit Messwiederholung mit der Effektstärke Eta Quadrat (η^2^) berechnet, wobei ab 0,01 ein kleiner, ab 0,06 ein mittlerer und ab 0,14 ein großer Effekt gilt. Wenn sich die Gruppen zu t1 signifikant unterschieden, wurde der Wert zu t1 als Kovariate in das Modell aufgenommen. Dies war bei allen Parametern bis auf die Subskala „Leben mit RA“ der Fall. Um die Wechselwirkung der Krankheitsdauer (≤6 Monate und >6 Monate) und des Bildungsniveaus auf den Parameter „PKQ-Original“ zu berechnen, wurden diese einzeln als zweiter Faktor in die ANOVA aufgenommen. Der Chi-Quadrat-Test (Χ^2^) wurde verwendet, um Unterschiede in der Häufigkeitsverteilung zu vergleichen. Als Signifikanzniveau wurde 0,05 festgelegt. Die statistischen Analysen wurden mittels SPSS durchgeführt.

## Ergebnisse

### Stichprobenbeschreibung

Die Gesamtstichprobe umfasst nach Ausschluss eines Drop-outs in der KG insgesamt 61 Teilnehmer mit RA, welche vom Juli 2016 bis Oktober 2018 sukzessiv in die Studie eingeschlossen wurden sowie den PKQ-Fragebogen zu t1 und t2 ausfüllten. Die soziodemografischen und krankheitsspezifischen Daten sind in der Tab. 3 dargestellt. Das Durchschnittsalter in der IG betrug 53 ± 12 Jahre, 91 % waren weiblich und in der KG 60 ± 15 Jahre mit einem Anteil von 62 % Frauen. Die durchschnittliche Krankheitsdauer betrug bei der IG 3,4 ± 1,7 Monate (KG 0,7 ± 1,1) bzw. bei Patienten mit Behandlungswechsel 23,7 ± 34,3 Monate (KG 83,8 ± 86,0).

**Table 3:** 

	Interventionsgruppe (IG) StruPI-RA	Kontrollgruppe (KG) Ratgeber der Deutschen Rheumaliga	*p*-Wert IG vs. KG	Hedge’s g
Geschlecht (%)				
Frauen	91 (*n* = 29)	62 (*n* = 18)	0,008 (Χ^2^)	–
Männer	9 (*n* = 3)	38 (*n* = 11)	–	–
*Alter *(Jahre) ± SD (Spannweite)				
Frauen	52,2 ± 11,9 (23–75)	61,7 ± 14,4 (25–76)	0,019	0,77
Männer	62,3 ± 5,8 (59–69)	56,9 ± 15,3 (29–76)	0,585	–
Bildungslevel (%)				
Gruppe 1^a^	81 (*n* = 26)	78 (*n* = 21)	0,741 (Χ^2^)	–
Gruppe 2^b^	19 (*n* = 6)	22 (*n* = 6)	–	–
Krankheitsdauer seit Diagnose (Ø Monate ± SD)				
Neuerkrankungen (Krankheitsdauer ≤6 Monate)	60; 3,4 ± 1,7 (1–6) (*n* = 18)	62; 0,7 ± 1,1 (0–3) (*n* = 18)	0,871 (Χ^2^)	–
			<0,001	1,99
Therapiewechsel (Krankheitsdauer >6 Monate)	40; 23,7 ± 34,3 (7–103) (*n* = 12)	38; 83,8 ± 86,0 (8–302) (*n* = 11)	0,005	1,02
Druckschmerzhafte Gelenke (TJC28) (Ø Anzahl ± SD) (*n*)	3,47 ± 4,27 (*n* = 30)	7,31 ± 6,69 (*n* = 29)	0,004	0,71
Geschwollene Gelenke (SJC28) (Ø Anzahl ± SD) (*n*)	2,43 ± 2,99 (*n* = 30)	5,34 ± 7,07 (*n* = 29)	0,067	–
*Visuelle Analog Skala(VAS)-Arzt* (Ø mm ± SD) (*n*)	30,5 ± 27,9 (*n* = 26)	60,6 ± 19,0 (*n* = 13)	0,002	1,25
*Blutkörperchensenkungsgeschwindigkeit (BSG)* (Ø mm/1 h ± SD) (*n*)	26,0 ± 20,9 (*n* = 28)	33,9 ± 19,7 (*n* = 28)	0,075	–
*C-reaktives Protein (CRP)* (Ø mg/dl ± SD) (*n*)	1,00 ± 1,61 (*n* = 29)	2,36 ± 3,12 (*n* = 29)	0,167	–
Disease Activity Score (DAS28) (Ø Pkte ± SD) (*n*)	3,62 ± 1,31 (*n* = 26)	4,83 ± 1,62 (*n* = 18)	0,010	0,88

*SD* Standard Deviation

^a^Bildungsniveau Gr. 1: keinen Abschluss, Hauptschul‑/Volksschulabschluss, Realschulabschluss, polytechnische Oberschule

^b^Bildungsniveau Gr. 2: Hochschulreife, Abitur

Die IG und KG unterscheiden sich in der Zusammensetzung etwas hinsichtlich Geschlecht und Alter sowie krankheitsspezifischer Parametern. So war in der KG der Anteil der männlichen Teilnehmer höher, die Schwere der Erkrankung höher (VAS, TJC28 und SJC28) sowie die Krankheitsdauer bei Therapiewechsel länger.

### Krankheitsspezifischer Wissenserwerb

**Figure 2:**
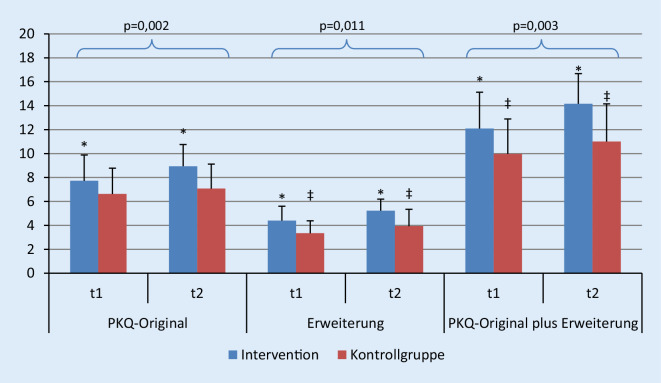

Die Teilnahme an StruPI-RA geht in der IG mit einer signifikanten Verbesserung des krankheitsspezifischen Wissens im Gruppen- und Zeitvergleich im Vergleich zur KG einher (*p* = 0,002, η^2^ = 0,15 im PKQ-Original; *p* = 0,011, η^2^ = 0,11 in Erweiterung; *p* = 0,003, η^2^ = 0,15 im PKQ-Original plus Erweiterung) (Abb. 2 und im Zusatzmaterial online).

Sowohl der PKQ-Originalwert der Patienten der IG (d = 0,61) als auch der Wert des Erweiterungsfragebogens (d = 0,76) und der PKQ-Originalwert plus Erweiterung (d = 0,74) verbessern sich signifikant (s. Abb. 2 und im Zusatzmaterial online). Im Gegensatz dazu steigen in der KG nur die Werte für die Fragebogenerweiterung (d = 0,48) und der PKQ-Originalwert plus Erweiterung (d = 0,34) an (Abb. 2).

Es zeigte sich kein signifikanter Einfluss der Krankheitsdauer (*p* = 0,847) und des Bildungsniveaus (*p* = 0,594).

Von den Subskalen zeigte nur die zur „Therapie“ im Gruppen- und Zeitvergleich eine signifikante Verbesserung der IG im Vergleich zur KG (Tab. 4).

**Table 4:** 

	StruPI-RA (*n* = 32)			Kontrollgruppe (*n* = 29)			ANOVA Zeit × Gruppe	
(M ± SD)	t1	t2	Cohen’s d	t1	t2	Cohen’s d	*p*-Wert	η^2^
Subskala Krankheitsbild und Diagnose (5 Fragen)	3,13 ± 1,21*	3,81 ± 1,18*	0,57	2,31 ± 1,04*	2,83 ± 1,34*	0,43	0,089	–
Subskala Therapie (8 Fragen)	5,97 ± 1,69*	6,84 ± 1,35*	0,57	5,07 ± 1,67	5,28 ± 1,69	–	0,002	0,16
Subskala Leben mit RA (5 Fragen)	3,00 ± 1,08*	3,50 ± 0,80*	0,53	2,59 ± 1,02	2,90 ± 1,26	–	0,481	–

Bei allen Parametern bis auf die Subskala „Leben mit RA“ wurde der Wert zu t1 als Kovariate in das Modell aufgenommen

*M* Mean, *SD* Standard Deviation

* *p* < 0,05 t1 vs. t2

## Diskussion

Diese Evaluation hatte das Ziel, erste Effekte einer Patientenedukation mittels der strukturierten Patienteninformation (StruPI) in der ambulanten Versorgung von Patienten mit RA zu untersuchen. Die Ergebnisse dieser kontrollierten Studie zeigen, dass mit Teilnahme an StruPI-RA gegenüber einer reinen Krankheitsinformation mittels einer ausführlichen Patientenbroschüre der Deutschen Rheumaliga ein erhöhtes krankheitsspezifisches Wissen zugunsten der Interventionsgruppe einhergeht. Mit dieser Studie konnte erstmals die Wirksamkeit eines ambulanten deutschen Schulungsprogramms, gemessen am PKQ-Fragebogen für Patienten mit früher RA oder nach Therapiewechsel, belegt werden. Die Veränderung war dabei unabhängig vom Bildungsstand und der Krankheitsdauer.

Sowohl die IG als auch die KG haben sich in ihrem Wissensstand verbessert, der Anstieg in der IG (Teilnahme an StruPI-RA) war aber höher und signifikant. Die Verbesserung in der KG erklärt sich durch das Lesen des PKQ zu t1 und somit die Kenntnis der relevanten Fragen in der Studie hinsichtlich des Wissenserwerbs. Auch die Lektüre des Ratgebers der Rheumaliga dürfte mit einer Erhöhung des Wissens verbunden sein. Der Anstieg des krankheitsbezogenen Wissens sowohl in der IG und KG lassen sich insbesondere durch die Subskala „Therapie“ erklären: D. h. die StruPI-RA-Patienten wiesen insbesondere hinsichtlich der Inhalte des Moduls 2, in dem die therapiebezogenen Aspekte vertieft werden, einen Wissenszuwachs auf.

Die Ergebnisse der Studie von Hennell [16], die mittels des Fragebogens PKQ eine deutliche Verbesserung des krankheitsspezifischen Wissens bei Patienten in England mit früher Rheumatoider Arthritis nach Durchführung eines vergleichbaren ambulanten Schulungsprogramms nachwies (*p* < 0,001, von 7,8 auf 9,7), konnten in dieser Studie bestätigt werden (*p* < 0,05, von 7,7 auf 8,9).

### Diskussion der methodischen Einschränkungen (Limitationen)

Eine Limitation der Studie ist die relativ kleine Fallzahl. Gleichwohl war die Stichprobe mit *n* = 61 Patienten groß genug, um statistisch signifikante Unterschiede im Gruppen- und Zeitvergleich nachzuweisen. Als Hindernisse für die Durchführung von StruPI-RA und damit auch als ursächlich für die geringen Patientenzahlen im Rahmen dieser Studie wurden am häufigsten der „Mangel an Zeit für den Abschluss der *Train-the-Trainer*-Ausbildung“ und der „Mangel an Entschädigung für die Schulungsseminare“ bezeichnet [20, 26]. Dies ist dem zeitlichen Aufwand in der IG, dem vorgeschalteten *Train-the-Trainer*-Seminar und der starken Arbeitsverdichtung im Alltag rheumatologischer Schwerpunktpraxen geschuldet. Nichtdestotrotz konnte erstmalig eine ambulante Patientenschulung im kassenärztlichen Versorgungsbereich erfolgreich umgesetzt und evaluiert werden.

Die Rekrutierung unter klinischen Alltagsbedingungen, bei der für die IG stärker motiviert und selektiert wurde für die Teilnahme an StruPI-RA, wohingegen die Patienten für die KG sukzessive eingeschlossen wurden, führte teilweise zu signifikanten Unterschieden in den soziodemografischen und krankheitsspezifischen Daten. Um dies auszugleichen, wurden diese jeweils als Kovariate in das statistische Modell aufgenommen.

Der PKQ hat sich als nützliches Werkzeug erwiesen, um den Wissenserwerb von Patienten mit früher Arthritis zu bewerten. Es ist nach Ergebnissen der Vorstudien einfach auszufüllen und erscheint für die Patienten im ambulanten Bereich in Deutschland akzeptabel [20]. Das erworbene Wissen ist Voraussetzung für fundierte Entscheidungen über Behandlungsmöglichkeiten, die Erhöhung des Selbstmanagements und das Patienten-Empowerment. Dies sollte sich langfristig positiv auf den Krankheitsverlauf, die Funktions- und Arbeitsfähigkeit sowie die Lebensqualität der Patienten auswirken. Ein Schulungsprogramm wie StruPi-RA könnte einen Beitrag leisten, um Ängste und Fragen hinsichtlich Diagnose und Therapie so früh wie möglich zu thematisieren und notwendige Adaptionen hinsichtlich Medikamenteneinnahme und Lebensstiländerungen zu befördern.

In weiteren Analysen der StruPI-RA-Evaluation sollen die Ergebnisse dieses prospektiven Kontrollgruppendesigns mit einer höheren Fallzahl und nach 3 (t3) und 6 Monaten (t4) überprüft werden. Insbesondere Unterschiede im Wissenserwerb hinsichtlich Geschlecht, Alter und Krankheitsschwere sind dabei zu untersuchen. Darüber hinaus ist es sinnvoll, prospektiv die oben genannten Outcome-Parameter zu erheben.

Dieser erste Nachweis eines Effektes von StruPI-RA ist Voraussetzung für eine Implementierung von StruPI-RA oder einer darauf aufgebauten Patientenschulung in die Versorgung von RA-Patienten [24]. Besonders zu betonen ist hierbei, dass es sich um eine überschaubare (3-mal 90 min), ambulant und gemeinsam von Rheumatologen und rheumatologischen Fachassistenten durchzuführende Patientenschulung handelt, die zu Beginn der Erkrankung oder bei einem notwendigen Therapiewechsel den RA-Patienten angeboten werden kann. Eine solche Implementierung einer Patientenschulung wird inzwischen auch in nationalen und internationalen Leitlinien als ein wichtiges Element der umfassenden Behandlung von RA-Patienten gefordert [25].

Moderne Patientenschulung dient dem „Empowerment“ von Patienten. Während traditionellerweise unter Schulung meist Wissensvermittlung verstanden wurde, stellen aktuelle Konzepte Handlungskompetenzen und motivationale Faktoren in den Mittelpunkt [8]. Den Teilnehmern sollen dabei Strategien und Fertigkeiten zur Verfügung gestellt werden, um informierte Entscheidungen und Selbstmanagement hinsichtlich Gesundheit und Lebensstil vornehmen zu können. Entsprechend hat sich die Didaktik weg vom Frontalvortrag hin zu einem interaktiven, teilnehmerorientierten Vorgehen gewandelt [8].

In einem erweiterten Schulungsprogramm wurde vor Kurzem, basierend auf einem modularen Rahmenprogramm [23, 24] und den Erfahrungen mit StruPI-RA, eine 5‑stündige Patientenschulung für den stationären und ambulanten Sektor entwickelt (StruPS), bei der rheumatologische und psychologische Kompetenz kombiniert angeboten werden. Darin wurde StruPI-RA als das Basismodul für die RA aufgenommen. In einer Evaluation dieses Programmes mit einer relativ großen Patientenzahl in einem Wartegruppendesign konnte dabei gezeigt werden, dass sogar 3 Monate nach der Schulung die teilnehmenden Patienten mehr Wissen und eine größere Gesundheitskompetenz hatten als die Kontrollgruppe [14]. Damit stehen den Patienten mit RA jetzt 2 evaluierte Programme zu Verfügung, die aufeinander abgestimmt in einer Version von 3‑mal 90 min im ambulanten Bereich (StruPI-RA) und mit 5 h im ambulanten, akutstationären und rehabilitativen Bereich (StruPS-RA) durchgeführt werden können. Durch die Flexibilität und inhaltliche Kongruenz dieser Programme sind Patienteninformation und -schulung auch über Versorgungsgrenzen hinweg möglich. Als besonderer Vorteil ist ein großes Maß an Flexibilität durch den modularen Aufbau beider aufeinander abgestimmter Programme hervorzuheben.

## Fazit für die Praxis

- Im Sinne einer umfassenden Versorgung von Patienten mit einer frühen RA und nach Therapiewechsel sind ambulante Informations- und Schulungsprogramme ein zentraler Bestandteil in der multimodalen Therapie.
- StruPI-RA hat sich als niederschwellige, strukturierte und interaktive Intervention als umsetzbar erwiesen.
- Die Evaluation von StruPI-RA im ambulanten Versorgungsalltag zeigt positive Effekte hinsichtlich des krankheitsspezifischen Wissenserwerbs. Die Patienten erleben StruPI-RA als positiv und profitieren hinsichtlich des erworbenen Krankheitswissens.
- StruPI-RA kann so Patientenwissen, Therapiesicherheit und Therapietreue und damit wahrscheinlich auch den Therapieerfolg verbessern helfen.
- Es erscheint sinnvoll, die Implementierung dieses funktionierenden Programms in den Versorgungsalltag weiter voranzutreiben.

## Caption Electronic Supplementary Material


