# Supplementary material for: Disease-related knowledge acquisition through structured patient information in rheumatoid arthritis (StruPI-RA): First results of the StruPI-RA study in Germany
Source: Z Rheumatol. 2020 Sep 14;80(4):364–72. [Article in German] doi: 10.1007/s00393-020-00871-7 (PMC8096752; doi:10.1007/s00393-020-00871-7)
Supplement: Supplementary file 1 [file 393_2020_871_MOESM1_ESM.pdf]

Zusatzmaterial

Patient Knowledge Questionnaire (PKQ) sowie Erweiterungsfragen

Fragen zum Krankheitsbild

**1. Was beschreibt die rheumatoide Arthritis am besten?**	**trifft zu**
a. Bei der rheumatoiden Arthritis handelt es sich um die Entzündung nur eines Gelenks.	○
b. Rheumatoide Arthritis betrifft meist die großen Gelenke.	○
c. Rheumatoide Arthritis ist eine entzündliche Systemerkrankung. Der Verlauf ist meist lebenslang, individuell sehr unterschiedlich und wellenförmig (schubweise).	○
d. Bei der rheumatoiden Arthritis handelt es sich um eine akute Gelenkentzündung.	○
e. Weiß nicht	○

**2. Wie kommt es zur rheumatoiden Arthritis?**	**trifft zu**
a. Rheumatoide Arthritis ist ausschließlich durch das Wetter bedingt.	○
b. Rheumatoide Arthritis entsteht vorwiegend durch schlechte Ernährung.	○
c. Viele Faktoren tragen zur Entstehung von rheumatoider Arthritis bei.	○
d. Verletzungen sind die überwiegende Ursache für rheumatoide Arthritis.	○
e. Weiß nicht	○

**3. Was beschreibt die Symptome eines akuten Krankheitsschubes der rheumatoiden Arthritis am besten?**	**trifft zu**
a. Schmerzen, Steifheit und Schwellung eines oder mehrerer Gelenke	○
b. Schmerzende(s) Gelenk(e)	○
c. Schmerzen und Steifheit	○
d. Grippeähnliche Symptome	○
e. Weiß nicht	○

**4. Womit kann die Krankheitsaktivität überprüft werden?**	**trifft zu**
a. Leberwerte	○
b. DAS 28 (Fragebogen/Score zu Gelenkbefund und Schmerzen)	○
c. Fragebogen zur Lebensqualität	○
d. Ultraschall	○
e. Weiß nicht	○

**5. Mit welchen beiden Bluttests kann überprüft werden wie aktiv die Arthritis ist?**	**trifft zu**
a. Blutkörperchensenkungsgeschwindigkeit (BSG) und CRP	○
b. Großes Blutbild und Plasmaviskosität	○
c. Großes Blutbild und CRP	○
d. Plasmaviskosität und GPT	○
e. Weiß nicht	○

Fragen zur Therapie

6. Welche direkte Wirkung haben krankheitsmodifizierende Antirheumatika (Basistherapie, DMARDs wie z.B. Methotrexat, Sulfasalazin, Leflunomid)?	
	**trifft zu**
a. Sie lindern die Schmerzen	○
b. Das Fortschreiten der Erkrankung wird durch die Einnahme verlangsamt.	○
c. Sie verbessern die Mobilität/Beweglichkeit	○
d. Verbesserung des Schlafes	○
e. Weiß nicht	○

7. Wie wirken sog. nicht-steroidale Antirheumatika (NSAR) wie z.B. Diclofenac, Ibuprofen, Naproxen, Coxibe (mehrere Antwortmöglichkeiten treffen zu)?	
	**trifft zu**
a. Schlaffördernd	○
b. Schmerzlindernd	○
c. Entzündungshemmend	○
d. Verlangsamen das Fortschreiten der Erkrankung	○
e. Weiß nicht	○

8. Was würden Sie tun, wenn Sie eine Hautveränderung bemerken?	
	**trifft zu**
a. Nicht weiter beachten	○
b. Selbst Medikamente kaufen (z.B. eine Creme)	○
c. Mit dem behandelnden Rheumatologen, der rheumatologischen Fachassistenz oder meinem Hausarzt sprechen	○
d. Die Einnahme aller meiner Medikamente beenden	○
e. Weiß nicht	○

9. Wieso ist eine weiterführende, regelmäßige Kontrolle der Blutwerte wichtig?	
	**trifft zu**
a. Um Schmerzparameter zu überprüfen	○
b. Um Nebenwirkungen (der Medikamente) und den weiteren Verlauf der Erkrankung über die Blutwerte zu erkennen	○
c. Um Schäden an den Gelenken festzustellen	○
d. Um die Mobilität zu fördern	○
e. Weiß nicht	○

10. Wann würden Sie ein Kühlkissen nutzen?	
	**trifft zu**
a. Bei einem gesunden Gelenk	○
b. Bei einem Hautausschlag	○
c. An einem heißen Tag	○
d. Bei einem warmen, geschwollenen Gelenk	○
e. Weiß nicht	○

11. Was würden Sie bei einem entzündlichen, schmerzhaften Knie machen?	
	**trifft zu**
a. Das Bein hoch halten und mit einem Kühlkissen kühlen	○
b. Das Bein auf einem Stuhl/Kissen hochlagern und das Knie mit warmen Umschlägen	○

behandeln.

c. Den ganzen Tag ins Bett legen ○
d. Das Bein auf einem Stuhl/Kissen hochlagern und das Knie mit einem Kühlkissen kühlen. ○
e. Weiß nicht ○

12. Welche der folgenden Aussagen zum Thema „Kortison“ ist richtig?

	trifft zu
a. Kortison bewirkt eine schnelle und starke Besserung von Entzündungsprozessen, Gelenkschwellungen und –schmerzen.	○
b. Kortison beeinflusst den Krankheitsverlauf in jedem Fall negativ.	○
c. Bereits die kurzzeitige Einnahme von Kortison führt schon zu Osteoporose.	○
d. Kortison hat keine Nebenwirkungen.	○
e. Weiß nicht	○

13. Welche Medikamentengruppe wird zur Behandlung einer rheumatoiden Arthritis eingesetzt (mehrere Antwortmöglichkeiten treffen zu)?

	trifft zu
a. Antikoagulantien („Blutverdünner“), z.B. Marcumar	○
b. Basismedikamente, z.B. Methotrexat (MTX) oder Biologika, z.B. Humira	○
c. Antibiotika	○
d. NSAR (nicht-steroidale Antirheumatika)	○
e. Weiß nicht	○

Fragen zum Leben mit rheumatoider Arthritis

14. Wie oft sollten Sie ein Heimtrainingsprogramm durchführen?

	trifft zu
a. 1 mal pro Monat	○
b. 1 mal pro Woche	○
c. mindestens 1 mal pro Tag	○
d. Nie	○
e. Weiß nicht	○

15. Wie können Sie am besten mit Ihren Kräften im Alltag haushalten?

	trifft zu
a. So lange wie möglich weiter arbeiten	○
b. Die Heizung in der Wohnung runterdrehen	○
c. Arbeiten ggf. im Sitzen anstatt im Stehen durchführen	○
d. Nicht über die Schmerzgrenze gehen	○
e. Weiß nicht	○

16. Welche der folgenden Möglichkeiten ist die Beste für Ihre Alltagsroutine?

	trifft zu
a. Alle Aufgaben am Morgen erledigen, damit Sie sich den Rest des Tages ausruhen können.	○
b. Regelmäße Ruhephasen über den gesamten Tag einplanen	○
c. Andere bitten, so viel wie möglich für Sie zu übernehmen	○
d. Trotz Schmerz weiterarbeiten	○
e. Weiß nicht	○

17. Welche sportlichen Aktivitäten sind mit rheumatoider Arthritis besonders gut für Sie geeignet?	
	**trifft zu**
a. Marathonlauf	○
b. Ski fahren/Snowboard fahren	○
c. Sanfter Ausdauersport (z.B. Schwimmen und Radfahren)	○
d. Squash/Tennis	○
e. Weiß nicht	○

18. Mit welchen kurzfristigen Maßnahmen können Sie ihre Schmerzen lindern?	
	**trifft zu**
a. Die Schmerzen ignorieren	○
b. Immer wieder bis an die Belastungsgrenze gehen	○
c. Sich nicht mehr bewegen und warten, bis der Schmerz nachlässt	○
d. Durch Ablenkung (z.B. ein Spaziergang, Reden, Kartenspielen)	○
e. Weiß nicht	○

Ergänzung zu Abbildung 2: Rohdaten

	**StruPI-RA (n=32)**			**Kontrollgruppe (n=29)**			**ANOVA Zeit x Gruppe**	
(MW ± SD)	**t1**	**t2**	**Cohen‘s d***	**t1**	**t2**	**Cohen‘s d**	**p-Wert**	**η²**
PKQ-Original (12 Fragen)	7,7 ± 2,2*	8,9 ± 1,8*	0,61	6,6 ± 2,2	7,1 ± 2,1		**0,002**	0,15
Erweiterung (6 Fragen)	4,4 ± 1,2*	5,22 ± 0,97*	0,76	3,34 ± 1,04*	3,93 ± 1,41*	0,48	**0,011**	0,11
PKQ-Original plus Erweiterung (18 Fragen)	12,09 ± 3,04*	14,16 ± 2,52*	0,74	9,97 ± 2,92*	11,00 ± 3,15*	0,34	**0,003**	0,15

* Die Grenzen für die Größe des Effekts liegen bei 0,01 (kleiner Effekt), 0,06 (mittlerer Effekt) und 0,14 (großer Effekt).
